# The associations between dietary pattern of chronic obstructive pulmonary disease patients and depression: a cross-sectional study

**DOI:** 10.1186/s12890-020-01383-5

**Published:** 2021-01-06

**Authors:** Fahimeh Dinparast, Akbar Sharifi, Sara Moradi, Maedeh Alipour, Beitullah Alipour

**Affiliations:** 1 Department of Nutrition, Tabriz University of Medical Sciences, Tabriz, Islamic Republic of Iran; 2 Tuberculosis and Lung Diseases Research Center, Tabriz University of Medical Sciences, Tabriz, Islamic Republic of Iran; 3 Faculty of Medicine, Tabriz University of Medical Sciences, Tabriz, Islamic Republic of Iran; 4 Department of Community Nutrition, Faculty of Nutrition, Tabriz University of Medical Sciences, Daneshgah St, Tabriz, 041‐33357581 Islamic Republic of Iran

**Keywords:** Chronic obstructive pulmonary disease, Depression, FFQ, Dietary patterns, FEV1/FVC

## Abstract

**Background:**

Chronic obstructive pulmonary disease (COPD) is a common lung disease during middle age which one of its complications is depression. Depression is considered one of the major causes of severe disability worldwide. One of the factors that affect the severity and incidence of this disease is a lifestyle, especially dietary pattern. On the other hand, some studies showed the relationship between dietary patterns and depression. The present study aims to investigate the dietary patterns of people with chronic obstructive pulmonary disease and its association with depression.

**Methods:**

The present cross-sectional study was performed on 220 patients (mean ± SD age = 54.58 ± 5.08) with chronic obstructive pulmonary disease (56.6% men, 43.4% women) from Tabriz, Iran. Questionnaires of general information, food frequency, Beck depression and physical activity were completed. The dominant dietary patterns were determined by factor analysis, and their relationship with depression was discussed by regression analysis.

**Results:**

Three dominant dietary patterns were identified as healthy, unhealthy, and mixed dietary patterns. An inverse relationship was found between healthy and mixed dietary patterns with depression. There is no meaningful connection between unhealthy dietary patterns and depression. Depression had a significant inverse relationship with physical activity. There was no relationship between dietary patterns and Forced Expiratory Volume for 1 s (FEV1) and Forced Vital Capacity (FVC) criteria. A positive and significant relationship was observed between mixed dietary patterns with FEV1/FVC.

**Conclusion:**

Inverse relationships exist between healthy dietary patterns and depression in patients with COPD, and improves the function of the lungs. Further studies are needed to show the exact relationship between diet and COPD depression.

## Background

Chronic obstructive pulmonary disease (COPD) is one of the most common chronic, progressive and irreversible diseases which is characterized by limited airflow in small airways, chronic inflammation, and destruction of the lung parenchyma [1]. COPD is currently the fourth leading cause of death in the United States and estimates showed the disease will grow to be the world's third-leading cause of death due to population growth, aging, and greater exposure to risk factors by 2030 [2]. The prevalence of COPD in Tehran, Iran was 9.2% in 2015 [3]. One of the complications of COPD is depression, which is usually accompanied by anxiety [4]. Depression is an affective-cognitive disorder characterized by decreased social interactions, sadness, and lack of motivation, enjoyment, and productivity [5]. The relationship between COPD and depression is probably bilateral as depression can be a cause and a consequence of the disease, simultaneously. However, the accurate mechanism of the association between COPD and depression or anxiety has not yet been established [6]. The prevalence of anxiety and depression in patients with COPD is higher than those with other chronic diseases such as hypertension, diabetes, cancer, or musculoskeletal disorders which probably due to decreased respiratory, physical, and functional strength, drug dependence, and frequent hospitalizations [7, 8].

Studies showed many patients with chronic diseases have mental disorders such as depression due to the long-term nature of the disease, and their impact on their quality of life [9–11]. Also, previous studies demonstrated dietary patterns in chronic disease such as hypertension, and cancers were associated with quality of life and as a healthier diet, as improved quality of life [12–14]. Besides, evidence suggests that nutrition has an impact on mental health [15]. For example, diet is associated with inflammation, oxidative stress, and brain function which all of these physiological factors are involved in developing depression [16, 17]. The most common approach until now was examining the relationship between diet and disease based on certain nutrients or foods [18, 19]. But the complex interaction between nutrients or foods fails to provide a relationship between diet and mental health in pulmonary disease [20]. Therefore, the importance of analyzing dietary patterns in this topic is emphasized [21–23] especially in developing countries [24, 25]. As the diet may affect COPD and depression separately, the present study aims to investigate the dietary patterns of people with COPD and its association with depression.

## Methods

This cross-sectional study was performed on 220 people with COPD, included participants referred to the Lung Research center in Tabriz University of Medical Sciences were supervised by a clinical specialist (April 2018 to April 2019). Inclusion criteria were having FEV1 < 80% and FEV1/FVC < 70% in Spirometry, ages 35–60, no specific diet, don’t use of a psychotropic drug or any experience of an accident during the past six months, and willingness to contribute to the study. Exclusion criteria were major changes in diet, physical activity, or medications during the study.

**Fig. 1:**
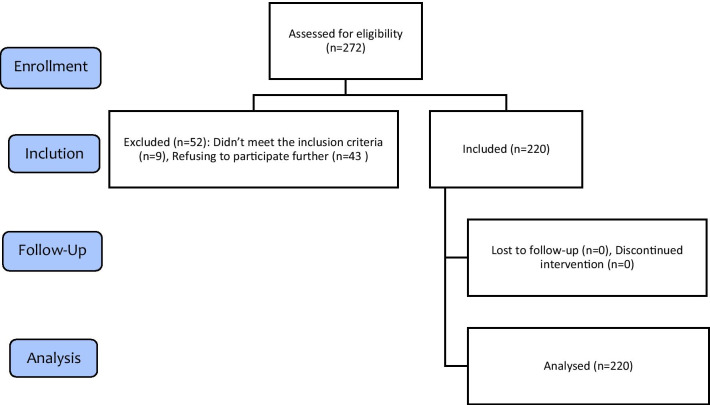
Flow diagram

The flow diagram was shown in Fig. 1.

General demographic data such as age, sex, marital status, education, smoking, medication, comorbidities were collected through a general information questionnaire. The weight was measured with a Seca scale (d = 100 g) to evaluate anthropometric data. The height was also measured using a tape meter with an accuracy of one-tenth of a centimeter as standard. Body mass index (BMI) was calculated as the body mass divided by the square of the body height in units of kg/m2. All information on spirometric criteria such as FEV1, FVC, and FEV1/FVC was collected using the latest data recorded in the patient's hospital files.

Beck Depression Inventory-II is a self-scored questionnaire for measuring depression severity in psychiatric patients as well as the normal population. It has 21 four-option multiple-choice questions based on a score of zero to three. This questionnaire identifies different degrees of depression from mild to severe and measures the physical, behavioral, and cognitive symptoms of depression [26]. Passer et al. evaluated Beck's Depression Inventory and reported that its internal consistency (Cronbach's alpha) was 0.92, its validity is 0.75 with one-week interval and its correlation coefficients vary from 0.30 to 0.76. Five types of validity such as content, concurrent, construct, discriminant, and factor were assessed with this test, all of which indicated the desirable performance of this tool in measuring depression severity [27]. The amount of daily physical activity in terms of the metabolic equivalent of 1 h per day (MET.h/day) was assessed by a valid and reliable international IPAQ physical activity questionnaire [28, 29].

The amount of nutritional intake of the past year was assessed by using a reliable semi-quantitative food frequency questionnaire including 132 items [30]. The subjects were asked to indicate the frequency of consuming each food in the questionnaire by considering the standard amount of the day, week, month, or year. The data of the questionnaire were analyzed as consumption per month. 132 foods were first grouped into 40 predefined food groups based on their nutrient similarities to determine the dietary patterns.

Factor analysis was used to identify dietary patterns. Varimax rotation was used to obtain a simple matrix with better interpretability and extract the desired nutritional patterns. Changing the curve of the scree plot was used to determine the dietary pattern factors. Finally, three dominant dietary patterns were extracted which accounted for 22.6% of the total variance. In this study, the load factor was considered more than 0.2 for determining the food groups in each meal. Kolmogorov–Smirnov test was used to evaluate the normality of distributing quantitative variables. Independent t-test or Mann–Whitney U test were employed to compare the quantitative variables and the Chi-squared test was used to compare the qualitative variables. Ranking regression was used to determine the relationship between dietary patterns and depression degree by adjusting confounding variables and a level of significance less than 0.05 was considered.

## Results

220 participants included in the study and no one lost to fallow-up. The mean age of participants was 54.58 ± 5.08 (56.6% men and 43.4% women). Demographic characteristics of participants showed in Table 1. The mean score of depression among participants was 22 ± 9.5. The prevalence of depression was 90.8%, of which 24.5% had a mild type, 42.7% had moderate level and 23.6% had severe depression. Tables 1 and 2 demonstrate the basal characteristics of participants.

**Table 1:** Results related to the general profile of individuals (n = 220)

Variable		Number (%)
Sex	Men	124 (56.6)
	Women	96 (43.4)
Marital status	Married	212 (96.4)
	Single	8 (3.6)
Level of education	Illiterate	141 (63.3)
	Undergraduate	60 (27.1)
	Graduate	15 (6.8)
	Academic	4 (1.8)
Occupation	Employee	5 (2.3)
	Worker	13 (5.9)
	Unemployed	13 (5.9)
	Housekeeper	91 (42.2)
	Others	94 (42.5)
Smoking	Non-smoker	153 (69.5)
	Smoker	67 (30.5)
Comorbidity	Family history of illness	154 (69.7)
	Hypertension	25 (11.3)
	Other diseases	41 (19)
Age (y) (Mean ± SD)		54.58 (± 5.08)

**Table 2:** Results related to anthropometric characteristics, depression score and distribution of subjects according to BMI status, physical activity, degree of depression and respiratory function test

Variable	Total n = 220	Men (n = 124)	Women (n = 96)	P*
	Mean ± SD			
Height (cm)	160.85 ± 12.10	166.77 ± 81.7	153 ± 23.7	< 0.05
Weight (kg)	70.30 ± 13.63	72.15 ± 14.22	67.91 ± 12.5	< 0.02
BMI (kg/m^2^)	27 ± 4.72	25.87 ± 4.54	28.64 ± 4.50	< 0.05
depression score	22 ± 9.58	23 ± 9.95	22.38 ± 9.09	0.41
*Pulmonary function testing*				
FEV1	57.18 ± 13.77	55.1 ± 13.8	59.7 ± 13.2	0.014
FVC	82.28 ± 16.7	79.7 ± 17.6	85.6 ± 15.5	0.009
FEV1/FVC	62.23 ± 9.34	61.6 ± 8.6	63 ± 8.6	0.238
	N (%)			
*BMI*				
Low = < 18/5	7 (3.2)	6 (2.7)	1 (0.5)	< 0.05
Normal = 18.5–24.9	70 (31.8)	52 (23.6)	18(8.2)	
Overweight = 25–29.9	79 (35.9)	40 (18.2)	39 (17.7)	
Obesity = > 30	64 (29.1)	26 (11.8)	38 (17.3)	
*Level of physical activity (MET/day)*				
low < 600	177 (80.5)	90 (40.9)	87 (39.5)	< 0.05
medium (2999–600)	32 (14.5)	24 (10.9)	8 (3.6)	
high = > 3000	(5)11	10 (4.5)	1 (0.5)	
*Depression score*				
No depression	20 (9.1)	12 (5.5)	8 (3.6)	0.40
Mild	54 (24.5)	32 (14.5)	(10)22	
Moderate	97 (42.7)	56 (25.5)	38 (17.3)	
Severe	52 (23.6)	24 (10.9)	28 (12.7)	

The values represent the frequency of individuals in each group and the numbers in parentheses. Chi-squared test was used to compare the two groups. *Independent T test

BMI, Body mass index; FEV1, Forced expiratory volume in 1 s; FVC, Forced vital capacity

**Fig. 2:**
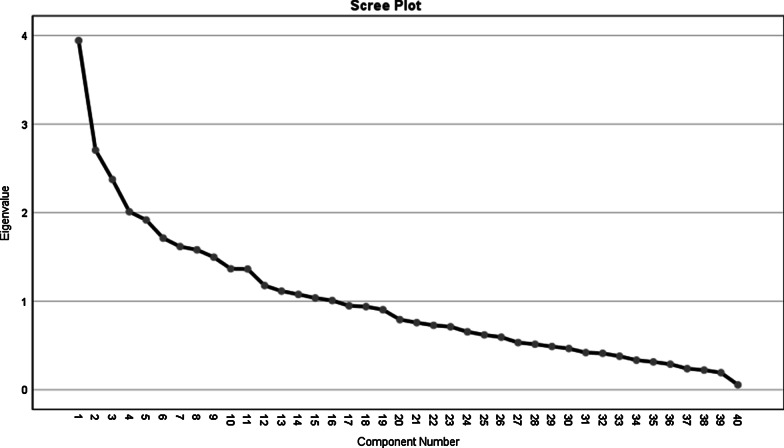
Determining the number of dietary patterns factors

Table 3 showed the Factor loadings of food groups in identified dietary patterns. Factor load values less than 0.2 have been omitted. Kaiser—Meyer—Olkin measure of sampling adequacy (KMO) was 0.544, and Bartlett’s test of sphericity was 0.001. By using the factor analysis method three dominant dietary patterns were identified in the subjects. Patterns were named based on food groups as the healthy eating pattern, unhealthy dietary pattern, and mixed dietary pattern. The healthy eating pattern consists of fruits, yellow vegetables, garlic, tomatoes, other vegetables, poultry, doogh, grains, nuts, potatoes, low-fat dairy products (percent variance = 9.8). The unhealthy dietary pattern consists of pastries, pickles, drinks, processed meats, snacks, coffee, offal, pizza, butter, refined cereals and high fat dairy (percent variance = 6.8). Mixed dietary pattern consists of dressings, dried fruit, mayonnaise, saturated fat, whole grains, red meat and cruciferous vegetables (percent variance = 5.9). Figure 2 determines the number of dietary pattern factors.

**Table 3:** Factor loadings of food groups in identified dietary patterns

Food groups	Mixed dietary pattern	Healthy eating pattern	Unhealthy dietary pattern
Dressings	0.713	–	–
Dried fruit	0.711	–	–
Vegetable oil	0.637	–	–
Mayonnaise	0.540	–	–
Saturated fat	− 0.512	–	–
Cruciferous vegetables	0.422	–	–
Whole grains	0.266	–	–
Red meat	− 0.233	–	–
Fruits	–	0.635	–
Garlic	–	0.609	–
Other vegetable	–	0.525	–
Tomato	–	0.383	–
Poultry	–	0.331	–
Doogh	–	0.330	–
Grains	–	0.328	–
Nuts	–	0.310	–
Yellow vegetable	–	0.300	–
Potato	–	0.294	–
Juice	–	0.218	–
Pastries	–	–	0.612
Coca	–	–	0.602
Processed meats	–	–	0.566
Meal	–	–	0.500
Coffee	–	–	0.462
Pickles	–	–	0.403
Butter	–	–	0.333
Offal	–	–	0.261
Pizza	–	–	0.247
Refined cereals	–	–	-0.237
High fat dairy	–	–	0.205
Percent variance	5.9	9.8	6.8

Factor load values less than 0.2 have been omitted for ease of use

Kaiser–Meyer–Olkin Measure of Sampling Adequacy (KMO) = 0.544

Bartlett’s test of sphericity = 0.001

Table 4 presents the results of the ordinal regression test adjusted for confounding factors (including age, sex, education, occupation, smoking, medication, comorbidities, and BMI). There was a significant inverse relationship between healthy (0.001) and mixed dietary patterns and depression (p = 0.032). There was no relationship between unhealthy dietary patterns and depression (p = 0.299).

**Table 4:** Relationship between dietary patterns and depression

Variable	Unadjusted B (95% CI)	P*	Adjusted B (95% CI)	P*
Depression				
healthy dietary pattern	− 0.31 (− 0.54, − 0.08)	0.007	− 0.41 (− 0.66, − 0.16)	0.001
unhealthy dietary pattern	0.12 (− 0.10, 0.35)	0.288	0.12 (− 0.11, 0.37)	0.299
mixed dietary pattern	− 0.26 (− 0.49, − 0.03)	0.230	− 0.27 (− 0.52, − 0.02)	0.032

^*^Ordinal Regression Test on two unadjusted and adjusted for confounding factors (including age, sex, education, occupation, smoking, medication, comorbidities, and BMI)

There was no significant relationship between dietary patterns with FEV1 and FVC. No relationship was found between FEV1/FVC criterion and healthy and unhealthy dietary patterns. However, this criterion was significantly in line with the mixed dietary pattern, i.e. adhering to the mixed pattern was associated with increases the FEV1/FVC ratio (Table 5).

**Table 5:** Relationship between dietary patterns and respiratory testing

Variable	B (CI 95%)	P*
FEV1 (%)		
Healthy pattern	0.9 (− 0.6, 2.6)	0.24
Unhealthy pattern	− 0.06 (− 2.3, 1.0)	0.43
Mixed pattern	0.9 (− 0.7, 2.5)	0.29
FVC (%)		
Healthy pattern	0.2 (− 1.8, 2.3)	0.79
Unhealthy pattern	1.4 (− 0.5, 3.5)	0.15
Mixed pattern	0.3 (− 1.7, 2.4)	0.71
(%) FEV1/FVC		
Healthy pattern	− 0.1 (− 1.2, 1.0)	0.83
Unhealthy pattern	− 0.4 (− 1.5,0.6)	0.43
Mixed pattern	1.2 (0.1, 2.3)	0.03

^*^Trend test

FEV1, Forced expiratory volume in 1 s; FVC, Forced vital capacity

## Discussion

This study implied three types of healthy, unhealthy, and mixed dietary patterns in the subjects. There are many similarities between the patterns obtained in this study and the dietary patterns extracted from other studies on chronic disease [31, 32]. As our study, along with other previous studies, vegetables, fruits, grains, low-fat dairy and olives contributed the most to a healthy diet and processed meats, red meat, beverages, pastries, fried potatoes, sugar, saturated fat, and refined grains have the largest portion of unhealthy dietary patterns [33–35]. The prudent diet, which included vegetables, fruits, seafood, poultry, whole grains, legume, and low-fat dairy. The Western dietary pattern of high consumption of refined cereals, processed meats, fried foods, and red meat is almost similar to the healthy western patterns found in the present study [36].

Shaheen et al. in 2010 showed A “prudent” dietary pattern may protect against impaired lung function and COPD, especially in male smokers [37]. Also, Ardestani et al. demonstrate adherence to DASH as a healthy diet which is low in patients with COPD [38]. Although, In this study, fish groups were not included in a healthy dietary patterns like Rezazadeh and Rashidkhani’s which may indicate low fish consumption in the population study [35].

The prevalence of depression in the present study was high (90%), which was in line with the study conducted by Adeli et al. (83.3%)[39]. Although, Laurin suggested that mental disorders in patients with COPD were about 49% [40]. There are several reasons for the differences in results, including differences in the methods of measuring depression, the number of participants, and the type of study.

Depression is a common mental disorder which nutrition plays a significant role in its occurrence, prevention to treatment. In the present study, a significant inverse relationship was found between healthy and mixed dietary patterns and depression after adjusting for confounding factors. This dietary pattern was similar by the results in Iran [34, 36]. In another study, After adjusting the effects of age, socioeconomic status, education, and health behaviors, the traditional dietary pattern was associated with a reduced risk of depression, whereas there was no correlation between the Western dietary pattern and depression [41]. In the study conducted by Akbaraly et al. [22] the complete dietary pattern consisting of all fruits and vegetables and the processed diet included sweetened desserts, fried foods, processed meats, refined cereals and high-fat dairy. Individuals who consumed a complete dietary pattern had a lower risk of depression whereas a higher risk of depression was observed in those who consumed processed food after adjusting for confounding variables. In the study conducted by Nanneri et al. in Japan, a healthy Japanese diet characterized by a high intake of fruits and vegetables, fungi and soy products which associated with a decrease in symptoms of depression [23]. On the other hand, Diepnal et al. in 2014 found a high carbohydrate diet could reduce the risk of depression [42].

According to previous studies, some nutrients such as omega-3, vitamins B12, B6, E, D, folate, magnesium, zinc, iron, copper, calcium and amino acid tryptophan are effective in treating depression [43, 44]. All foods found in healthy dietary patterns include vegetables, fruits, dairy, kernels, olive oil and its seeds, fish and whole grains rich with the mentioned nutrients, so effect on depression symptoms [45]. One study demonstrated The inverse relationship between dietary pattern and depression is due to fish and red meat in this model which are a rich source of Methionine that play a key role in producing Adenosyl methionine, a neurotransmitter such as Serotonin. Reducing this neurotransmitter is associated with disorders such as stress, depression, obsession and anger [46]. Although, our study showed different findings, due to a low intake of fish.

A positive relationship was found between the mixed dietary pattern and the FEV1 ratio in the present study. The association between dietary patterns and pulmonary function represented that adherence to a healthy diet pattern was associated with high FEV1, and FVC in both sexes. Men had also higher scores for FEV1/FVC and lower COPD prevalence in the upper quartile of the healthy dietary pattern. In agreement with our study, this association was higher in smokers than non-smokers [37]. In another study, a healthy dietary pattern (containing high amounts of fruits and vegetables, fish and nuts) and a carbohydrate-rich pattern were also positively correlated with FEV1 values [47]. The results of the Brigham study suggested that a healthy diet pattern in which the consumption of vegetables, fruits, fish, poultry and whole grains is high reduces COPD symptoms such as cough, and improves lung function. In return, the western diet, which was characterized by high consumption of refined cereals, processed red meat, fries, eggs, and soda, was associated with a higher risk of COPD [48].

## Strengths and limitations

The cross-sectional design of this study was its main limitation, which did not allow extracting the causal relationships between dietary patterns and depression. Other limitations of this study were failing to have a standard for determining the number of factors (patterns) in the factor analysis method and using a self-report tool for measuring depression. One of the limitations of this study was to measure depression with one scale. Maybe cross-check with another questionnaire to estimate depression with a more accurate scale. Another limitation is used FFQ for evaluation food intake, without cross-checking with 3 days food records, and not taking into consideration people's nutritional behaviors (pattern, time, and number of snacks and meals). The strength of this study was to assess dietary patterns in patients with COPD in Iran for the first time with this sample size.

## Conclusion

This study demonstrated the healthy dietary pattern in contrast to the unhealthy diet, associated with a low prevalence of depression, and improves the function of the lungs in patients with COPD. Further studies can be used to highlight the consumption of healthy and mixed dietary patterns by considering the high prevalence of depression among patients with COPD and also the importance of nutrition in preventing and treating illness and controlling the severity of depression.

## Data Availability

The data are available from the corresponding author, upon request.

## Abbreviations

AAT: Alpha Antitrypsin
ATS: American Thoracic Society
BMI: Body Mass Index
CES-D: Center for Epidemiologic Studies Depression Scale
COPD: Chronic Obstructive Pulmonary Disease
CRP: C-reactive protein
DASH: Dietary Approaches to Stop Hypertension
DSM-IV: Diagnostic and Statistical Manual of Mental Disorders, Fourth Edition
FEV 1: Forced Expiratory Volume for 1 s
FFQ: Food Frequency Questionnaire
FVC: Forced Vital Capacity
GOLD: Global Initiative for Chronic Obstructive Lung Disease
GMS: Geriatric Mental State Schedule
HADS: Hospital Anxiety and Depression Scale
ICAM-1: Intercellular Adhesion Molecule 1
IL-6: Interleukin 6
IPAQ: International Physical Activity Questionnaire
KIHD: Kuopio Ischemic Heart Disease
MDI: Metered-dose Inhaler
MESA: Multi-Ethnic Study of Atherosclerosis
MET: Metabolic Equivalent for Task
PCA: Principal Component Analysis
PREDIMED: Prevention on Mediterranean Diet
QOL: Quality of Life Scale
SD: Standard Deviation
SPSS: Statistical Package for Social Science
TNF: Tumor Necrosis Factor
